# 1. INTRODUCTION

**DOI:** 10.1093/eurpub/ckac127

**Published:** 2022-10-25

After two years of virtual-only conferences, we are very pleased that Berlin 2022 will be in person again. That our network has a high interest in meeting in person to intensify the networking was clearly reflected in the new record of abstract and workshop submissions. Our dedicated International Scientific Committee had the difficult job of scoring all the submissions and we are—as always—very grateful for their work, as their work ensures the high scientific quality of the conference programme.

This supplement to the European Journal of Public Health contains the abstracts of presentations at the 15^th^ European Public Health (EPH) Conference, held in Berlin from 9-12 November and includes abstracts for presentations for the main part of the conference (plenary sessions, oral presentations, workshops and poster displays).

For the EPH Conference 2022, we received a record 2.371 abstracts including 186 workshops. All abstracts were scored by the members of the International Scientific Committee (ISC) of the EPH Conference. The ISC 2022 consisted of 132 public health experts from 31 countries and was chaired by Prof Verena Vogt. To ensure scientific quality without bias, each abstract was scored by on average 3,81 reviewers, each workshop by 3,95. We are extremely grateful to the ISC members who scored in 2022. These members are listed below.

The abstracts were scored on a scale of 0–7. The average score of the single abstracts was 4.517 (Virtual 2021–4.381). The highest score was 6.667 and the lowest 1.000. The average score of workshops was 4.546 (Virtual 2021–5.050). The highest score was 6.667 and the lowest 2.000. Only the highest scoring abstracts are accepted for the EPH Conference. The rejection rate for the single abstracts was 30% and for workshops, the rejection rate was 35%. The EPH Conference Executive Board decided on 10 June 2022 on cut-off points for workshops, oral presentations and poster presentations, resulting in an excellent selection of workshops and presentations, as showcased in this abstract supplement.

**Table:** 

Workshops	average score of 4.333 or higher were accepted
Oral presentations (8-minute presentation)	average score of 5.750 or higher were accepted
Pitch presentations (5-minute presentation)	average score of 5.333 or higher were accepted
E-Poster walks (3-minute presentation)	average score of 5.100 or higher were accepted
E-Poster displays (throughout the programme)	average score of 4.200 or higher were accepted

Following these decisions, abstracts were grouped in sessions, sessions in tracks and tracks were linked to the contents of the plenary programme. The programme for EPH Conference 2022 is organised in 16 thematic tracks, that mix workshops and oral sessions.

The 16 thematic tracks are: The posters are on display throughout the whole conference.

- Strengthening health systems: improving population health
- Preparing for the unexpected: lessons learned from Covid-19
- European public health/Food and nutrition
- Chronic diseases
- Environment, climate and health
- Digital health and communication
- Health data, information and assessments
- Health determinants and health inequalities
- Health services and welfare systems
- Health promotion, health literacy, behavioural insights
- Health workforce, training and practice
- Infectious diseases, preparedness and vaccines
- Maternal, child and adolescent public health
- Mental health/Migration and minority health/LGBTQI+health
- Policy, politics and public health
- Public health monitoring, reporting and foresight

As always, the International Scientific Committee members greatly enjoyed reading the submissions, many of them stating that it is a great exercise to learn about new developments in their field of expertise. We hope that you will find this volume equally interesting, and even more so the actual presentations, which promise to be of high quality yet again.

Looking forward to welcoming you all in Berlin,

Prof Reinhard Busse, Chair of the 15^th^ European Public Health Conference

Prof Verena Vogt, Chair of the International Scientific Committee

Dr Dineke Zeegers Paget, Strategic advisor EUPHA

International Scientific Committee, 2022, active — Prof Verena Vogt, Germany—Chair

— Dr Oboh Achioyamen, United Kingdom

— Prof Róza Ádány, Hungary

— Dr Nina Adelberger, Germany

— Prof Charles Agyemang, Netherlands

— Prof Kristina Alexanderson, Sweden

— Prof Peter Allebeck, Sweden

— Mrs Tatiana Alves, Portugal

— Prof Arja Aro, Finland

— Prof Kevin Balanda, Ireland

— Dr Gabriela Barbaglia, Spain

— Dr Andrea Barbara, Italy

— Dr Marleen Bekker, Netherlands

— Prof Fabrizio Bert, Italy

— Dr Regien Biesma-Blanco, Netherlands

— Prof Johan Bilsen, Belgium

— Dr Christopher Birt, United Kingdom

— Dr Henrik Bøggild, Denmark

— Dr Richard Bränström, Sweden

— Prof Ute Bültmann, Netherlands

— Prof Genc Burazeri, Albania

— Prof Reinhard Busse, Germany

— Dr Stefan Buttigieg, Malta

— Dr John M Cachia, Malta

— Prof Stefano Capolongo, Italy

— Dr Marion Carey, Australia

— Paloma Carrillo-Santisteve, Belgium

— Dr Evangelia Chrysikou, United Kingdom

— Prof Nesrin Cilingiroglu, Turkey

— Dr Thomas Classen, Germany

— Prof Nadav Davidovitch, Israel

— Prof Judith de Jong, Netherlands

— Prof Corrado De Vito, Italy

— Prof Chiara de Waure, Italy

— Prof George Delclos, United States

— Prof Marie-Luise Dierks, Germany

— Prof Julia Dratva, Switzerland

— Natalie Durbeej, Sweden

— Mr Gilles Dussault, Portugal

— Prof Paksoy Erbaydar, Turkey

— Prof Carlo Favaretti, Italy

— Prof Rainer Fehr, Germany

— Prof Patricia Fitzpatrick, Ireland

— Dr Silvia Florescu, Romania

— Prof Anders Foldspang, Denmark

— Dr Kimberley Foley, United Kingdom

— Mr Marthein Gaasbeek Janzen, Netherlands

— Prof Maria Gańczak, Poland

— Mr Manuel García Goñi, Spain

— Prof Amandine Garde, United Kingdom

— Dr Abby Gold, United States

— Prof Beatriz González-Valcarcel, Spain

— Prof Marcus Grant, United Kingdom

— Prof Peter Groenewegen, Netherlands

— Prof Giuseppe Grosso, Italy

— Dr Stefano Guicciardi, Italy

— Prof Catherine Hayes, Ireland

— Dr Gunnel Hensing, Sweden

— Dr Thomas Hone, United Kingdom

— Prof Kate Hunt, United Kingdom

— Mr Damir Ivankovic, Netherlands

— Dr Danielle Jansen, Netherlands

— Prof Marta Cecilia Jaramillo-Mejia, Colombia

— Prof Marija Jevtic, Serbia

— Prof Ramune Kalediene, Lithuania

— Prof Ilona Koupil, Sweden

— Prof Allan Krasnik, Denmark

— Dr Ellen Kuhlmann, Germany

— Dr Bernadette Kumar, Norway

— Prof Tobias Kurth, Germany

— Prof Giuseppe La Torre, Italy

— Prof Lucie Laflamme, Sweden

— Prof Alastair Leyland, United Kingdom

— Prof Jutta Lindert, Germany

— Dr Tomi Mäki-Opas, Finland

— Prof Julian Mamo, Malta

— Dr Marco Marchetti, Italy

— Prof Piedad Martin-Olmedo, Spain

— Ms Sara Mc Quinn, Ireland

— Dr Odile Mekel, Germany

— Prof Anjum Memon, United Kingdom

— Mrs Monika Mensing, Germany

— Dr Gabriele Messina, Italy

— Mrs Joana Moreno, Portugal

— Dr Anne Mosnier Mantel, France

— Dr Iveta Nagyova, Slovakia

— Prof Saoirse Nic Gabhainn, Ireland

— Dr Emer O'Connell, Ireland

— Prof Orkan Okan, Germany

— Dr Maria Papadakaki, Greece

— Prof Julian Perelman, Portugal

— Prof Ivan Perry, Ireland

— Dr Elena Petelos, Greece

— Dr Paulo Pinheiro, Germany

— Mr Klaus D. Pluemer, Germany

— Prof Ileana Prejbeanu, Romania

— Prof Ossi Rahkonen, Finland

— Dr Muhammad Aziz Rahman, Australia

— Dr Bina Ram, United Kingdom

— Prof Andrea Rebecchi, Italy

— Dr Sofia Ribeiro, Portugal

— Prof Eva Roos, Finland

— Dr Nicole Rosenkötter, Germany

— Prof Luís Saboga-Nunes, Portugal

— Mr Rui Santana, Portugal

— Prof João Vasco Santos, Portugal

— Prof Milena Šantrić Milićević, Serbia

— Prof Sonia Saxena, United Kingdom

— Prof Andreia SilvaCosta, Portugal

— Prof Luis Souza, Brazil

— Prof Anthony Staines, Ireland

— Prof Danijela Stimac Grbic, Croatia

— Prof Christiane Stock, Germany

— Prof Saverio Stranges, Canada

— Prof Birute Strukcinskiene, Lithuania

— Dr Amets Suess Schwend, Spain

— Prof Pernille Tanggaard Andersen, Denmark

— Dr Marius-Ionut Ungureanu, Romania

— Dr Arjan van der Star, Sweden

— Dr Aurélie Van Hoye, Ireland

— Ms Caroline Vass, United Kingdom

— Dr M Luisa Vázquez Navarrete, Spain

— Prof Arnoud Verhoeff, Netherlands

— Prof Susana Viegas, Portugal

— Prof Paolo Villari, Italy

— Prof Anne Vuillemin, France

— Dr Georgina Warner, Sweden

— Mr Greg Williams, United Kingdom

— Prof Silviya Yankulovska, Bulgaria

— Dr Dineke Zeegers Paget

